# Whole-Body Cold Stimulation Improves Cardiac Autonomic Control Independently of the Employed Temperature

**DOI:** 10.3390/jcm13247728

**Published:** 2024-12-18

**Authors:** Nadia Solaro, Luca Giovanelli, Laura Bianchi, Paolo Piterà, Federica Verme, Mara Malacarne, Massimo Pagani, Jacopo Maria Fontana, Paolo Capodaglio, Daniela Lucini

**Affiliations:** 1 Department of Statistics and Quantitative Methods, University of Milano-Bicocca, 20126 Milan, Italy; nadia.solaro@unimib.it; 2 BIOMETRA Department, University of Milan, 20129 Milan, Italy; luca.giovanelli@unimi.it (L.G.); mara.malacarne@unimi.it (M.M.); daniela.lucini@unimi.it (D.L.); 3 Servizio Neurofisiopatologia, IRCCS Istituto Auxologico Italiano, 28824 Piancavallo, VB, Italy; laurabianchitnfp@gmail.com; 4 Department of Neurosciences “Rita Levi Montalcini”, University of Turin, 10126 Turin, Italy; p.pitera@auxologico.it; 5 Laboratory of Clinical Neurobiology, IRCCS Istituto Auxologico Italiano, 28824 Piancavallo, VB, Italy; 6 Laboratory of Biomechanics, Rehabilitation and Ergonomics, IRCCS Istituto Auxologico Italiano, 28824 Piancavallo, VB, Italy; f.verme@auxologico.it; 7 Department of Biomedical, Surgical and Dental Sciences, University of Milano, 20122 Milano, Italy; massimo.paganiz@gmail.com (M.P.); p.capodaglio@auxologico.it (P.C.); 8 UOC Musculoskeletal and Metabolic Rehabilitation, IRCCS Istituto Auxologico Italiano, 20095 Milan, Italy; 9 Exercise Medicine Unit, IRCCS Istituto Auxologico Italiano, 20135 Milan, Italy

**Keywords:** autonomic nervous system, cryotherapy, heart rate variability, longitudinal data, nonparametric statistics, parasympathetic activity

## Abstract

**Background/Objectives**: The autonomic nervous system (ANS) is considered one of the mechanisms mediating the benefits of whole-body cold stimulation (WBC). Nevertheless, different treatment protocols, different methodologies employed to assess the ANS, and, in particular, difficulties in interpreting the numerous variables obtained represent important barriers to understanding the effects of WBC on the ANS. The present study aimed to explore the effects of WBC on cardiac autonomic control (CAR) as assessed using a single composite percentile-ranked proxy of autonomic balance (ANSI), considering two different WBC temperatures and the same WBC protocol. **Methods**: Heart rate variability (HRV) was employed to assess the ANS in 23 subjects with obesity who underwent 10 WBC sessions, studied before and after 2 min WBC at −55 °C (15 subjects) or 2 min WBC at −110 °C (8 subjects) both at the first session (T1) and the last one (T10). To overcome some important barriers in data interpretation and age/sex bias, we considered the Autonomic Nervous System Index (ANSI), a single composite percentile-ranked proxy of autonomic control. **Results**: We observed an improvement in CAR independently of the employed temperature. Both treatments, without distinction, caused a significant increase in the ANSI post-WBC treatment both at T1 and T10 and a significant betterment of the total power of the RR interval variability from pre- to post-treatment at T1 and overall from T1 to T10. **Conclusions**: WBC was capable of inducing an immediate change in the ANS control (pre- vs. post-treatment both at T1 and T10) and a long-term modulation in cardiac autonomic control (T1-pre vs. T10-pre).

## 1. Introduction

The use of cold for overall health can be tracked as far back as ancient Greece [1]. Nowadays, the use of cold as a tool capable of fostering well-being and managing pain is part of everyday practice [2,3]. Whole-body cold stimulation (WBC) has been seen to have rapid anti-inflammatory and antioxidant effects as well as modulate pain and the autonomic nervous system. According to a recent review on the safety and possible adverse events of WBC, when preceded by accurate medical screening, WBC is a safe and well-tolerated treatment featuring rare adverse events [4]. Presently, WBC has gained momentum as an adjuvant treatment in many conditions, from orthopaedic to neurological, metabolic, and autoimmune diseases [5]. WBC acts as an allostatic load to train our homeostatic systems. The beneficial effects seem to be mediated via the activation of the central nervous system and the consequent neurohumoral responses, which drive a cascade of changes in the endocrine, circulatory, neuromuscular, and immunological systems. Cold, in fact, represents a stress factor for the body that can stimulate an insulative autonomic response intended to limit heat loss (peripheral vasoconstriction), promote thermogenesis, and modulate nervous impulses, allowing adaptation to the external environment [5,6,7,8,9,10]. A key player in this thermogenic and adaptive response is brown adipose tissue (BAT), which is activated during WBC via the beta-adrenergic pathway of the sympathetic nervous system (SNS). BAT functions not only as a thermogenic organ but also as an endocrine and paracrine hub, releasing bioactive molecules that influence the CNS, autonomic nervous system (ANS), and peripheral organs [11].

Growing scientific evidence suggests that WBC might be considered not simply a symptomatic treatment for pain in inflammatory disorders but an adjuvant therapy for endocrine/metabolic diseases and dysautonomic conditions and for recovery from problems related to strenuous exercise training [5,12,13,14,15]. Cardiac autonomic regulation (CAR) may play a pivotal role among the mechanisms elicited by cold exposure [6,7,8,10,16,17,18,19,20,21].

The role of CAR as a mechanism involved in the pathogenesis of multiple diseases, such as arterial hypertension, coronary artery diseases, diabetes, obesity, cancer, stress, and other functional syndromes, is well established. Notably, the improvement of ANS control associated with many non-pharmacological interventions (exercise training, optimization of body mass composition, stress management, quitting smoke) contributes to rendering these interventions as strategies of paramount importance in preventing and treating many diseases [22]. WBC might be considered a further non-pharmacological intervention capable of fostering ANS modulation.

Several scientific publications are devoted to the study of ANS modulation with cryostimulation [7,8,10,23,24,25]. While there is a general agreement that there is an increase in variables considered markers of parasympathetic control [7,8,10,23,25] after WBC, a lack of consistency is observed regarding the employed WBC protocol, the methodology used to study ANS and/or different temperatures, the exposure duration, and the sessions’ frequency. Some papers refer simply to observed variations in heart rate (HR) or systolic arterial pressure (SAP) [26], while others employ heart rate variability (HRV) [7,8,10,23,24,25], a technique that may be performed using different methods. In particular, time domain analysis examines the variability of the RR intervals between consecutive heartbeats considering a sinus rhythm, while the frequency domain approach further assesses the power relationship of RR interval oscillations across two main frequency bands: low-frequency (LF) and high-frequency (HF, synchronous with breathing) bands. Specifically, this methodology may consider different mathematical approaches and the autoregressive one, which furnishes an analysis of LF and HF components both in absolute (coded in time units) and normalized units (nu) (addressing the relationship between the power of low- and high-frequency components) [27], is nowadays widely utilized in clinical settings [10]. While the main variable identified by the time domain approach (total variance, total power (TP) or RMSSD) is regarded as a marker of parasympathetic modulation, the frequency domain approach furnishes markers of both main sympathetic (LF) and parasympathetic (HF) prevalent modulations [27,28,29]. HRV is now de facto considered the gold standard methodology [30,31,32] for non-invasively studying the ANS. Nonetheless, the complexity of the analytical techniques and difficulty of interpretation, especially when mechanistically considering a single autonomic variable at a time, in addition to the well-known influence of age and sex on CAR, may act as barriers to the interpretation of the results, particularly during dynamic conditions, such as exercise [33,34] or cryostimulation. In order to overcome some of these barriers, given the fundamentally unitary nature of visceral neural regulation [35], we introduced an integrated, single composite Autonomic Nervous System Index (ANSI), which was designed as a percentile-ranked proxy for autonomic balance, with higher values indicating better autonomic control [36].

The aim of the present study was to explore, in a group of obese patients, the effects of WBC on cardiac autonomic control as assessed using a single composite percentile-ranked proxy of autonomic balance (ANSI), considering two different WBC temperatures and the same WBC protocol.

## 2. Materials and Methods

### 2.1. Participants

This study is part of an ongoing study on the overall effects of WBC in patients with obesity. In the present study, we specifically focused on the effects of the intervention on cardiac autonomic control.

Patients with obesity (body mass index, BMI > 35 kg/m^2^) were enrolled from adult inpatients at San Giuseppe Hospital, IRCCS Istituto Auxologico Italiano, Piancavallo, Verbania, Italy, specifically admitted for an obesity multidisciplinary rehabilitation program, which was the same for all enrolled patients. Between June 2021 and March 2023, 23 patients with obesity (18 women and 5 men), with a mean age of 45.48 ± 13.67 years, range 20–69 years, agreed to participate in this study. Severe psychiatric illness, acute respiratory disease, acute cardiovascular disease, active neoplasia or recent history of malignancy, hypertension not controlled by medical treatment, cold intolerance, claustrophobia, pregnancy, recent variations in usual medication, previous treatment with WBC, weight loss in the previous 3 months or body temperature above 37.5 °C were considered as exclusion criteria.

The 23 patients were consecutively evaluated for inclusion in the protocol and assigned to the following treatments:

- Treatment 1, in which $n_{1}=15$ subjects received 10 WBC sessions, once daily, at −55 °C for 2 min;
- Treatment 2, in which $n_{2}=8$ subjects received 10 WBC sessions, once daily, at −110 °C for 2 min.

**Figure 1:**
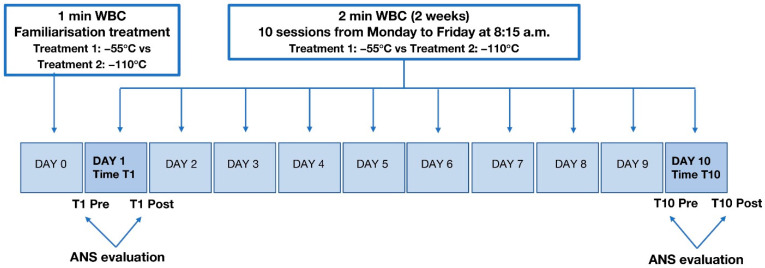
Protocol description.

A synthetic representation of the whole body cold-stimulation (WBC) sessions is provided in Figure 1. Patients underwent 10 sessions of WBC over a 2-week period (1 treatment per day, Monday through Friday, at 8:15 a.m., before exercise classes and physical therapy). Patients, healthcare providers administering the rehabilitation interventions, and laboratory personnel (excepting the researchers administering WBC) did not know the type of treatment administered, i.e., whether at −55 °C (treatment 1) or at −110 °C (treatment 2). The WBC session consisted of exposure to extremely cold, dry air at −55 °C or −110 °C for 2 min in a cryo-chamber (Artic, CryoScience, Rome, Italy). The familiarisation session (held the day before the first WBC session) consisted of 1 min of exposure to −55 °C or −110 °C, depending on the group assigned. Each session was carried out under the supervision of properly trained operators. Subjects were asked to remove glasses, contact lenses, and jewellery; to have dry skin; and to wear surgical mask, shorts or sweatpants, a light T-shirt or sports bra for women, mid-calf socks, clogs, gloves, and a head and ear cover; and to breathe naturally. Blood pressure was taken (Omron M3 HEM-7154-E, Omron Corporation, Kyoto, Japan) before and after each treatment session. Skin temperatures were recorded within 1 min before and after each WBC exposure using an infrared thermometer (Fluke 62 MAX+, Fluke Corporation, Everett, WA, USA) to verify the cooling effect of each WBC session on superficial skin temperature in four spots: calf and quadriceps, popliteal fossa, and upper body temperature (nape of the neck).

### 2.2. Autonomic Nervous System Evaluation

Blood pressure, HR, and HRV were recorded before and after the first WBC session (pre–post T1) and before and after the last WBC session (pre–post T10) (Figure 1).

An electrocardiogram (ECG) was continuously recorded over a minimum 5 min period using a three-channel ECG recorder (ADInstruments, Oxford, UK). Afterwards, subjects were asked to stand up unaided and stay upright for 5 min, during which recordings were maintained.

Acquired data were offline analysed at the Exercise Medicine Unit, IRCCS, Istituto Auxologico Italiano, Milan, using a custom-built software tool (HeartScope, version 2.0) that automatically provides a series of indices describing HRV in the time domain: RR interval (in msec) and RR interval variability (assessed as TP, i.e., variance, in msec^2^), taken as simple classifiers typical of vagal control [36,37,38]; and in the frequency domain, autoregressive spectral components both in the low frequency (LF, centre frequency ≈ 0.1 Hz) and in the high frequency (HF, centred with respiration, ≈0.25 Hz), assessed in msec^2^ as well as in nu. The stand–rest difference (Δ) in LFnu was also computed to approximately evaluate the effects of sympathetic activation produced by active standing. Arterial pressure was assessed using an electronic sphygmomanometer.

To simplify the clinical interpretation of the multitudes of HRV variables jointly considered, we developed a unitary autonomic index (Autonomic Nervous System Index, ANSI) [36] as a proxy for CAR. Such an index was built based on the main findings derived from factor analysis applied to HRV variables, whereby RR, RR interval variance, and ΔRR LFnu resulted in being highly representative of the cardiac autonomic information (considering amplitude and oscillatory code modalities) [29]. The procedure to construct the ANSI was as follows: First, to obtain variables adjusted for age and sex effects, the percentile rank (PR) transformation was applied to, respectively, RR, RR interval variance, and ΔRR LFnu within each age-by-sex class. Second, to obtain a unitary index, a radar plot (with a triangle shape) was built for each subject using the values of the three PR-transformed variables; then, the areas of the individual triangles were computed. Third, to have a normalized index, the PR transformation was applied again to the individual triangle areas. This way, the obtained ANSI can be regarded as a composite normalized indicator, free of sex and age bias, ranging in [0, 100], where higher values denote better autonomic control [39].

Patients were fully informed about the goal and methodology of the study, which was carried out in conformity with the Declaration of Helsinki of the World Medical Association and approved by the Ethics Committee of the IRCCS Istituto Auxologico Italiano (reference: 2021_05_18_14). Written and verbal informed consent was obtained from all patients.

### 2.3. Statistics

Descriptive statistics were computed for each studied variable (11 in all) concerning the ANS evaluation (i.e., RR mean, RR TP, RR LFa, RR HFa, RR LFnu, RR HFnu, ΔRRLFnu, and ANSI) and arterial pressure (SAP and DAP) as median ± MAD (median absolute deviation) within each combination of treatment (−55 °C vs. −110 °C) by time (T1 vs. T10) and by phase (pre vs. post) (Figure 1). Then, to investigate the effects of WBC on cardiac autonomic control, statistical analyses were carried out with a twofold purpose:

(A) to evaluate whether, for each variable, there were (1) significant overall differences between the two treatments, i.e., significant treatment main effects; (2) significant time and phase main effects; or (3) significant interactions (of the first and the second order) concerning treatments, times, and phases;
(B) to measure the impact of the considered conditions on each variable.

To meet the above two purposes and because of the small sample size, we relied on the nonparametric, rank-based procedure for longitudinal data in factorial designs developed by Brunner and Puri [40] and Brunner et al. [41]. This procedure extends the parametric analysis of variance (ANOVA) of longitudinal data in factorial designs, usually performed through, e.g., mixed-effects linear models, to a nonparametric framework. This way, no distributional assumption is required on the variables, and, if present, outliers have no impact on the analysis results due to the rank-based approach. Based on the terminology by Brunner et al. [41], the protocol design in Figure 1 can be regarded as an F1-LD-F2 design, where F1 denotes the whole-plot factor “treatment” (with the two levels −55 °C and −110 °C), LD stands for longitudinal data, and F2 regards the two sub-plot factors “time” (with the two levels T1 and T10) and “phase” (with the two levels pre and post). Therefore, for each subject involved in the study, we considered the measurements taken on four occasions, i.e., twice (pre and post) at time T1 and twice (pre and post) at time T10, besides his/her treatment assignment (Figure 1).

Regarding objective (A), let $X_{gits}$ be a random variable describing the measure X collected in the treatment group g on patient i under the time–phase combination (t,s), with g=1, 2, $i=1,\ldots ,\;n_{g}$, t=1, 2, and s=1, 2. The starting point of this nonparametric approach is the assumption that $X_{gits}\sim F_{gts}(x)$, i.e., that $X_{gits}$ has (unknown) marginal distribution function $F_{gts}(x)$. Then, the hypotheses specified above can be expressed in terms of distribution functions, that is:

(1) Treatment main effects: $H_{0}^{F}(Treat.):{\bar{F}}_{1..}={\bar{F}}_{2..}$ where ${\bar{F}}_{g..}=\frac{1}{4}\sum_{t=1}^{2}\sum_{s=1}^{2}F_{gts}$ is the marginal distribution function of X in treatment g;
(2) Time and phase main effects: $H_{0}^{F}(Time):{\bar{F}}_{.1.}={\bar{F}}_{.2.}$, where ${\bar{F}}_{.t.}=\frac{1}{4}\sum_{g=1}^{2}\sum_{s=1}^{2}F_{gts}$ is the marginal distribution function of X at time t, and $H_{0}^{F}(Phase):{\bar{F}}_{..1}={\bar{F}}_{..2}$, where ${\bar{F}}_{..s}=\frac{1}{4}\sum_{g=1}^{2}\sum_{t=1}^{2}F_{gts}$ is the marginal distribution function of X at phase s;
(3) First-order interactions, e.g., time–phase interaction: $H_{0}^{F}\left(Time:Phase\right):{\bar{F}}_{.ts}={\bar{F}}_{.t.}+{\bar{F}}_{..s}-{\bar{F}}_{\ldots }$, for each t and s, where ${\bar{F}}_{.ts}=\frac{1}{2}\sum_{g=1}^{2}F_{gts}$ is the bivariate distribution function of X under the time–phase combination (t,s), and ${\bar{F}}_{\ldots }=\frac{1}{8}\sum_{g=1}^{2}\sum_{t=1}^{2}\sum_{s=1}^{2}F_{gts}$ is the marginal distribution function of X over all the conditions. The hypotheses $H_{0}^{F}\left(Treat.:Time\right)$ and $H_{0}^{F}\left(Treat.:Phase\right)$ can be written analogously. Second-order interaction: $H_{0}^{F}\left(Treat.:Time:Phase\right):F_{gts}={\bar{F}}_{gt.}+{\bar{F}}_{g.s}+{\bar{F}}_{.ts}-{\bar{F}}_{g..}-{\bar{F}}_{.t.}-{\bar{F}}_{..s}+{\bar{F}}_{\ldots }$, for each g, t, and s.

The null hypotheses are thus formulated similarly to the traditional parametric three-way ANOVA model but involving distribution functions instead of parameters. They are then tested through the so-called nonparametric ANOVA-type tests for the F1-LD-F2 design, which are based on the estimation of the unknown distribution functions through their corresponding empirical distribution functions, such as ${\hat{F}}_{gts}(x)$ for $F_{gts}\left(x\right)$ [40,41].

As far as concerns about objective (B), we applied the procedure by Brunner and Puri [40], based on the concept of stochastic tendency, to estimate the nonparametric relative effects of each condition c (e.g., the treatment–time–phase combination $\left(g,t,s\right)$, or the time–phase combination (t,s)) on the variables and obtained their 95% confidence intervals (CIs). These relative effects can be regarded as general expectations of the considered distribution functions, and, as such, they represent a synthesis value of theirs. To account for the different patients’ clinical conditions at the beginning of the study and treat them on an equal footing, we first adjusted the values of each variable X by computing the differences: $\tilde{X}=X-x_{T1,pre}$, where $x_{T1,pre}$ is the value of X observed under the combination “time = T1 and phase = pre”. Then, the relative effect $p_{c}$ of a generic condition c on the adjusted variable $\tilde{X}$ can be interpreted as the probability that a subject chosen at random from the whole subject set has a smaller value on $\tilde{X}$ than a subject randomly chosen from the condition c group. In practice, if $p_{c}>0.5$ ($p_{c}<0.5$), then values of $\tilde{X}$ from the condition c group tend to be larger (smaller) than in the whole subject set, while if $p_{c}=0.5$, then values of $\tilde{X}$ from the condition c group are neither larger nor smaller than the whole subject set. The relative effects were then regarded as measures of the impact that each considered condition c had on the studied variables. In particular, if the CI of $p_{c}$ contains the value 0.5, no significant difference is detected between the condition c group and the whole subject set.

The relative effects can also be compared among different conditions. If $c^{\prime }$ and $c^{\prime \prime }$ are two different conditions and $p_{c^{\prime }}<p_{c^{\prime \prime }}$ ($p_{c^{\prime }}>p_{c^{\prime \prime }}$), then condition $c^{\prime }$ has a smaller (greater) impact than $c^{\prime \prime }$, while if $p_{c^{\prime }}=p_{c^{\prime \prime }}$, there is no difference in terms of impact between the two conditions $c^{\prime }$ and $c^{\prime \prime }$. Therefore, if the CIs of $p_{c^{\prime \prime }}$ and $p_{c^{\prime \prime }}$ overlap, the two relative effects are not significantly different.

Since, in this study, the relative effects were estimated on the adjusted variables, they were more appropriately indicated as adjusted relative effects (AREs). Nonetheless, the rank-based procedure adopted for their estimation is precisely the one described in Brunner et al. [41].

Throughout the study, the nominal test significance level was set at 0.05. We performed the statistical analysis through the R software (R Core Team 2024) [42], with the following contributed packages: “nparLD” [43] for the nonparametric ANOVA-type tests and estimation of the AREs along with 95% CIs and “gplots” [44] for the profile plots of the AREs and their CIs.

## 3. Results

Table 1 displays the median ± MAD computed for the 11 studied variables within each treatment time–phase combination. It can be immediately noticed that patients in the treatment 2 group (−110 °C) at time T1, “pre” phase, have higher median values than the treatment 1 group (−55 °C) on all of the studied variables, except RR HFnu, SAP, and DAP, for which the median values are lower than in treatment 1 group. This tendency persists at time T10, “pre” phase. Patients assigned to the treatment 2 group seem then to be in better ANS condition than the treatment 1 group. The median values of the ANSI are, in fact, equal to 57.32 (±10.24) in the treatment 2 group vs. 39.95 (±15.14) in the treatment 1 group at time T1, “pre” phase, and equal to 62.10 (±17.12) vs. 46.55 (±13.63), respectively, at time T10, “pre” phase. The same trend can be seen in the “post” phase, where the ANSI median values are equal to 72.49 (±6.85) in the treatment 2 group vs. 63.76 (±7.53) in the treatment 1 group at time T1 and equal to 76.79 (±10.90) vs. 71.42 (±15.20), respectively, at time T10.

Nonetheless, by the performed nonparametric ANOVA-type tests (objective (A), Section Statistics), no significant results occur for the treatment effects, neither as main effects nor as interactions with time and/or phase, nor is the time–phase interaction significant on any considered variable (Table 2). Instead, the main effects of phase are significant in the case of RR Mean, RR TP, RR HFa, RR LFnu, RR HFnu, RR LFHF, and ANSI, while the main effects of time are significant only for RR TP. Therefore, overall, the two treatments have no significant difference in the studied variables, even if combined with the time and phase levels. In contrast, the obtained results suggest performing a more in-depth analysis of the main effects of the phase levels regardless of the type of treatment administered to patients.

To this end, Table 3 displays the AREs estimated for the two-phase levels, “pre” and “post”, within the two levels, T1 and T0, of time. Following the description in Section Statistics, ARE $p_{c}$ provides, in this case, a measure of the impact of the specific time–phase condition c=(t,s) on the considered variables, i.e., without distinguishing the patients into the two treatment groups. Three remarks are worth making. First, for each time level, the estimated AREs of RR Mean, RR HFa, and ANSI increase when moving from the “pre” to the “post” phase. In particular, the associated CIs do not overlap, so the “pre” and “post” AREs are significantly different for each time level. In the case of RR TP, this fact occurs only at time T1, while in the case of RR LFnu, it occurs only at time T10 (with a decreasing trend). Second, concerning ANSI, it is interesting to note that at time T1, its estimated ARE is equal to 0.315 (CI: [0.271, 0.421]) in the “pre” phase and to 0.685 (CI: [0.579, 0.729]) in the “post” phase, while at time T10, the “pre” ARE is equal to 0.453 (CI: [0.425, 0.482]) and the “post” ARE is equal to 0.547 (CI: [0.518, 0.575]), respectively. Then, it seems that the WBC therapy has a stronger impact on ANSI in the pre–post comparison at time T1, i.e., at the beginning of the therapy, than T10, i.e., at the end. Nonetheless, the “pre” ARE at T10 (0.453) is estimated at a higher value than at T1 (0.315). Third, although RR HFnu and RR LFHF have associated significant main effects of phase (Table 2), their AREs have overlapping CIs over time and phase levels; thus, they are not significantly different in the various comparisons. These results do not contradict each other because the AREs can be regarded as synthesis values of a distribution function, while the ANOVA-type tests in Table 2 consider the entire distribution functions, not a single value.

**Figure 2:**
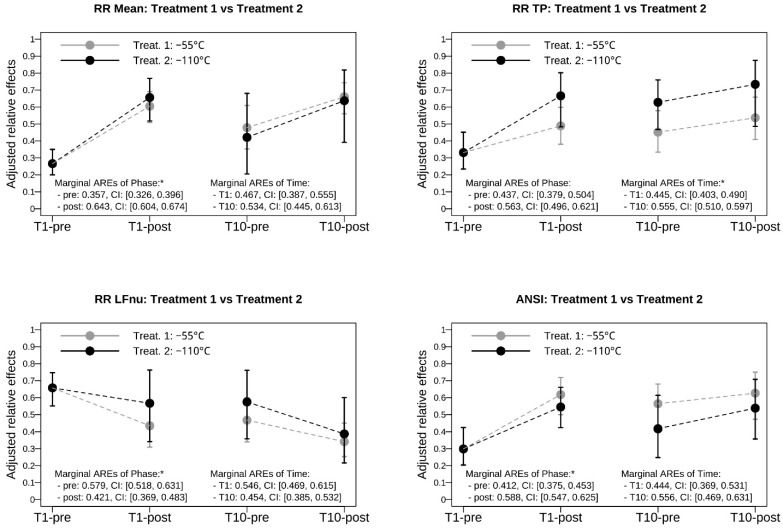
Profile plots of the nonparametric adjusted relative effects estimated for RR Mean, RR TP, RR LFnu, and ANSI of each combination of treatment (−55 °C vs. −110 °C), time (T1 vs. T10), and phase (pre vs. post). *Note*: The adjusted relative effects (AREs) considered in the profile plots are the estimated ${\hat{p}}_{gts}$ for each treatment-time-phase combination $\left(g,t,s\right).$ AREs estimated at time T1 in the “pre” phase, i.e., ${\hat{p}}_{g11}$ with g=1, 2 coincide over the two treatment groups because patients are put on the same footing after adjusting all of the variables with their initial values. Accordingly, the black point representing the estimated ARE of treatment 2 and its CI overlap perfectly with the grey point of treatment 1 and its CI, which is then hidden below the first. Moreover, in the panels, the estimated marginal AREs and their CIs are also reported for the two levels of phase: ${\hat{p}}_{..s}$, with s=1, 2, i.e., without considering the distinction in treatment and time; and the two levels of time: ${\hat{p}}_{.t.}$, with t=1, 2, i.e., without considering the distinction in treatment and phase. Significantly different marginal AREs at the 0.05 level are marked with an asterisk (i.e., their 95% CIs do not overlap).

Finally, to have a more complete picture of the obtained results, Figure 2 displays the profile plots of the AREs estimated for four selected adjusted variables, i.e., RR mean, RR TP, RR LFnu, and ANSI, within each treatment time–phase combination (numeric data for all of the adjusted variables are given in Supplemental Table S1). Results concerning the marginal effects of the phase and time levels, respectively, are also reported (numeric data for all of the adjusted variables are in Supplemental Tables S2 and S3). It can be noted that the CIs of AREs of the two treatments under the various time–phase combinations always overlap, in line with the non-significant differences between the two groups already pointed out (Table 2). Moreover, the increasing trend of AREs on RR mean, RR TP, and ANSI can be seen, as well as the decreasing trend of AREs on RR LFnu, when moving from the “pre” to the “post” phase, as already observed for the estimated time–phase AREs in Table 3. As a final remark, in the panels of Figure 2, the marginal time and phase AREs are also reported. It can be seen that time with its T1 and T10 levels has significant AREs on RR TP, while phase with its “pre” and “post” levels has significant AREs on RR mean, RR LFnu, and ANSI. In particular, the marginal impact of phase on ANSI is increasing: the “pre” ARE is equal to 0.412 (CI: [0.375, 0.453]) while the “post” ARE is equal to 0.588 (CI: [0.547, 0.625]), thus indicating that an improvement in cardiac autonomic control overall occurred immediately after the WBC session, regardless of the treatment type.

## 4. Discussion

In this paper, we observed, in patients with obesity who underwent WBC, an improvement in cardiac autonomic control independently of the employed temperature (−55 °C or −110 °C). In particular, we noticed that both treatments, without distinction, determined a significant increase of the unitary composite percentile-ranked proxy of overall cardiac autonomic control (ANSI) post-WBC treatment both at the first (T1) and the tenth session (T10) and a significant betterment of total power of RR interval variability, a marker of parasympathetic modulation, from pre- to post-treatment at session T1 (Table 3) and overall, from T1 to T10 (Figure 2). These data suggest that WBC was capable of inducing an immediate change in ANS control (pre- vs. post-treatment both at T1 and T10) and a long-term modulation in cardiac autonomic control (T1-pre vs. T10-pre).

During WBC, the entire body is briefly exposed to dry and very cold air in specially adapted cryo-chambers. Exposure to extreme cold stimulates cutaneous thermoreceptors, which drive the information to the thermoregulation centre in the hypothalamus [13]. Consequently, to maintain a constant core temperature, activation of the ANS regulatory centre triggers skin vasoconstriction and, hence, systolic and diastolic arterial pressure increases [7,16,17,18], resulting in a shift in blood volume towards the core [8,19,20]. The increase in central pressure, in turn, activates the baroreflex mechanisms responsible for shifting ANS control towards parasympathetic dominance [8] in view of restoring physiological homeostasis [10,19,21].

Another intriguing aspect to consider is the potential involvement of BAT in mediating the effects of WBC on the ANS, as assessed through HRV. BAT is activated by cold exposure via the SNS and plays a central role in thermogenesis and metabolic regulation [11]. This activation induces systemic responses that extend beyond thermogenesis, including endocrine and paracrine signalling, through the release of BATokines, such as fibroblast growth factor 21 and IL-6, which influence energy metabolism, inflammation, and neural activity [45]. The activation of BAT during WBC may contribute to the observed improvements in cardiac autonomic regulation by modulating SNS and parasympathetic nervous system (PNS) activity. The interplay among the BAT, ANS, CNS, SNS, and peripheral organs highlights the complex systemic effects of WBC. Cold-induced BAT activation likely interacts with hypothalamic pathways in the CNS, enhancing thermoregulatory and autonomic responses [46]. This interaction, coupled with increased baroreceptor activity due to blood volume redistribution towards the core, may amplify the parasympathetic tone, as reflected in HRV improvements. Peripheral organs also benefit from BATokine-mediated effects, such as improved metabolic and inflammatory profiles, further reinforcing systemic homeostasis [47].

Several studies showed that WBC might also have an influence on key inflammatory events, mitigating inflammatory response [9,48] and oxidative stress [9,49,50,51] with consequent local and systemic anti-inflammatory/antioxidant effects [12]. More specifically, WBC increased the concentration of the anti-inflammatory cytokine interleukin (IL)-10 and decreased both the pro-inflammatory cytokine IL-2 and chemokine IL-8 [12]. These pieces of evidence corroborate using WBC for treating many rheumatic [52] and metabolic diseases such as fibromyalgia and obesity [15]. Notably, autonomic, immunological, and metabolic controls are intercorrelated [53,54], and interventions capable of reducing fat mass and improving metabolic and immunological controls are also associated with an ANS improvement [31,38,55].

The positive local anti-inflammatory effects of WBC are also called into question to explain the use of this treatment to improve recovery after strenuous exercise [3,9] by minimizing sensations of delayed onset muscle soreness or exercise-induced muscle damage [14]. Moreover, some authors [14,24,56,57,58] hypothesized that using WBC before exercise execution could improve sports performance and well-being. WBC might positively modulate responses linked to exercise, such as testosterone and cortisol levels, ANS activation, and subsequent post-activation potentiation effects [14,24,56,58,59]. Acute heavy resistance exercise is associated with sympathetic overactivity [14,60,61], and the same ANS effect is provoked by abrupt and intense immersion into a cold environment [3,62]. According to some authors [14], this process could explain how WBC could favourably impact an athlete’s preparation and subsequent readiness. On the other hand, a WBC treatment after exercise might help to reduce sympathetic overactivity and improve parasympathetic activity. Notably, different WCB protocols, different temperatures, and different exposure times could elicit different ANS responses, calling for caution in data interpretation.

In the present paper, we applied a simple protocol (study of ANS control pre- and post-WBC treatment at the first session and the tenth one) considering two different temperatures: −110 °C (which is the widely applied temperature) and −55 °C (which is considered a light cold stimulus). Meta-analysis tried to address the issue of the effects of different WBC temperatures. Nevertheless, the complexity of employed protocols, the inhomogeneity of studies, and the methodologies employed to study ANS impeded clear conclusions [36]. We observed that both treatments were followed by an increase in ANSI, a marker of overall cardiac control. The statistical nature of this marker (see the following paragraph), built by integrating information derived from many HRV variables into a unitary proxy, permits a better unveiling of pieces of information that, if considered alone, might lead to discordant statistical analysis results, besides being affected by sex and age bias. Moreover, since the ANSI was built using a rank-based method, it is robust to the presence of outlying subjects, so statistical analyses performed on the ANSI are not sensitive to potential anomalies in the data. The observation that the ANS might be improved by a higher temperature (−55 °C) could facilitate wider use of WBC, particularly in those subjects with obesity or hypertension, characterized by sympathetic overactivity [38,55] and, hence, at increased cardiovascular risk. In fact, although WBC is considered a safe approach [4], some studies showed that cryogenic temperatures have a strong modulatory effect on the cardiovascular system, which, at least in some groups of patients, such as patients with hypertension, might be of concern [6,63]. Furthermore, the subjective perception of less cold might facilitate patients’ compliance.

The study of ANS in clinical settings using a simple, non-invasive methodology, such as HRV, may offer great chances. However, many methodological/interpretative [34] pitfalls in the last decades have hindered this opportunity. In particular, the common use of multiple indicators considered as indices of either vagal or sympathetic cardiac functionality (see, e.g., Malik et al. 1996 [28]) in the absence of an integrated approach may hamper a reliable inference on the underlying neural mechanisms. Additionally, the use of different protocols to study the ANS (for instance, studying it in resting conditions only or employing a dynamic approach also considering the changes induced by standing up), sex and age bias, the lack of reference tables, or limited study populations, pose many barriers to the wide use of this methodology and the interpretation of the derived variables. An example is offered by the important sex, age, and subject’s clinical condition influences on the more widely used HRV variable (the total variance of RR interval variability, in this paper defined as RR TP or in others as RMSSD), which is considered as a marker of parasympathetic cardiac modulation. Another example is offered by the interpretation of the “Low Frequency” (LF) or “High Frequency” (HF) components of the HRV using a frequency domain approach, particularly when employing an autoregressive approach to HRV considering a normalization between the two main spectral components detected. Since our group proposed the terminology “LF and HF” in 1986 [27], it is clear that these variables may not be considered a “measure” of sympathetic or parasympathetic controls.

Keeping all of these considerations in mind, we planned to obtain an index of autonomic cardiac regulation (ANSI) that would be free from sex and age bias, integrating in a single index proxy information on supine rest, standing up [36], and that could be simple to apply in a practical setting (clinical or physiological). Accordingly, the various cardiac autonomic markers were integrated into a unitary proxy using a radar plot. Furthermore, the use of the PR transformation permitted an individual evaluation within the reference population [36]. Summarising, the ANSI represents a single composite percentile-ranked proxy of autonomic balance, whereby higher values (from 0 to 100) indicate better autonomic control. Notably, the ANSI, which is built considering only variables derived from the HRV, correlated well with the Alpha Index (a marker of the overall gain of cardiac baroreflex sensitivity) [29].

Another important aspect that may underline the improvement of the ANS after WBC treatment is the subject’s report of pleasure and well-being after the treatment [14,15,64,65,66]. In this study, we observed that 2 min WBC treatment at both temperatures was associated with improved ANS control, as indicated by ANSI values at both the first and last sessions. Many studies on the effects of muscle and mental relaxation (well-known techniques to promote well-being) showed a clear improvement in ANS control, in particular, a shift from sympathetic towards parasympathetic modulation [67,68]. The betterment of ANS control observed immediately after the treatment and after ten sessions might contribute to the perception of well-being observed in both athletes and patients. Moreover, it might also take part in the rationale to use WBC treatment before sports competition to improve performance; in fact, well-being is a well-known factor regulating sports performance [69], and on the other hand, self-perceived stress and physical discomfort from prior training has been associated to reduced performance [70]. The role of WBC in improving well-being and cognitive deficits [9,70] opens new areas of intervention in different patients, such as patients with mental health problems. The report of pleasure and well-being after the treatment was also revealed by patients in our study. All patients tolerated the treatment well, and no adverse events of any kind or discomfort/stress were recorded, partly because all patients had a 1 min trial session to familiarize themselves with the treatment.

In conclusion, we observed that WBC caused an improvement in cardiac autonomic control immediately after treatment (pre- vs. post-treatment both at T1 and T10). Moreover, after 10 WBC sessions, parasympathetic control also seemed to be enhanced in basal conditions (T1-pre vs. T10-pre). This finding contributes to increasing the evidence that WBC might be considered an adjuvant therapy for those conditions characterized by an ANS imbalance. Moreover, it may mitigate inflammatory conditions and promote recovery from problems related to strenuous exercise training. Future research should focus on ensuring that appropriate protocols are employed, tailoring them to the subject/patient’s characteristics and needs.

## 5. Limitations

This study presents some important limitations. First, the study population involves a small group of patients with obesity enrolled to participate in a rehabilitation program to lose weight without a control group. Nevertheless, the rehabilitation program was the same in the two treatment groups (we already published elsewhere the data regarding the complete intervention [15]), and we observed modulation of the ANS immediately after treatment both at T1 and T10 utilizing a methodology to assess the ANS and analyse the data capable of unveiling changes even in a small group. The improvement in the total variance of the RR interval variability observed at baseline when comparing T1 and T10 might be due to rehabilitation per se. However, excluding some subjects from the rehabilitation program was not possible for ethical reasons. We also have to consider that a longer period (more than two weeks) of rehabilitation is required to significantly impact the ANS balance [71], particularly if the performed exercise is of light intensity, as in the study by Fontana et al. [72]. Secondly, this study employed a relatively short protocol of only two weeks (10 WBC sessions), limiting our ability to evaluate the long-term effects of WBC. Previous research has indicated that the effects of WBC may attenuate over time [23,25,64]. Nevertheless, we also observed a stronger impact of the WBC therapy on cardiac autonomic control (through the ANSI) at time T1 (the start of the therapy), compared to T10 (the end), suggesting an acute influence of WBC that warrants further exploration. Another limitation is the absence of an assessment of BAT activity in this study, including measurements of maximal thermogenic capacity via indirect calorimetry or sympathetic tone to BAT. While these techniques are highly informative, they are not easily accessible to all laboratories. Nevertheless, understanding potential alterations in BAT function would be essential for accurately interpreting the results in future studies.

This paper might offer scientific support for designing WBC interventions in a larger population, permitting statistical analyses of different groups characterized by different temperatures and sham interventions.

## Figures and Tables

**Table 1:** Summary statistics (median ± MAD) of the studied variables within the combinations of treatments (−55 °C vs. −110 °C), times (T1 vs. T10), and phases (pre vs. post).

		Treatment 1 (−55 °C)		Treatment 2 (−110 °C)	
Variables	Phase	Time = T1	Time = T10	Time = T1	Time = T10
RR Mean	Pre	875.31 ± 80.16	892.21 ± 45.84	929.74 ± 43.45	930.505 ± 92.67
	Post	923.72 ± 76.04	948.92 ± 65.02	974.46 ± 68.79	1029.685 ± 72.15
RR TP	Pre	852.31 ± 426.53	1414.62 ± 893.03	1379.03 ± 804.83	1956.37 ± 791.31
	Post	1282.07 ± 743.91	1509.17 ± 956.02	2765.07 ± 1502.47	2564.01 ± 706.52
RR LFa	Pre	185.03 ± 143.95	187.09 ± 156.68	317.31 ± 243.38	503.445 ± 231.27
	Post	112.49 ± 99.19	223.67 ± 198.94	755.115 ± 570.63	616.87 ± 378.13
RR HFa	Pre	137.57 ± 85.15	200.44 ± 104.79	578.13 ± 198.84	419.505 ± 272.11
	Post	219.45 ± 145.20	281.27 ± 223.14	811.41 ± 260.36	611.08 ± 264.38
RR LFnu	Pre	61.90 ± 17.88	35.39 ± 22.43	62.88 ± 10.95	59.18 ± 7.49
	Post	40.33 ± 15.87	39.45 ± 18.67	45.63 ± 8.74	46.84 ± 11.96
RR HFnu	Pre	35.09 ± 17.51	59.47 ± 17.64	30.71 ± 9.64	38.27 ± 11.16
	Post	49.80 ± 19.88	55.77 ± 22.45	43.08 ± 11.32	43.15 ± 9.61
RR LFHF	Pre	1.82 ± 1.48	0.55 ± 0.40	2.17 ± 1.30	1.60 ± 0.64
	Post	0.90 ± 0.67	0.71 ± 0.62	1.05 ± 0.35	1.06 ± 0.58
ΔRRLFnu	Pre	7.47 ± 8.90	20.75 ± 22.94	26.06 ± 27.03	26.36 ± 10.80
	Post	24.50 ± 18.46	22.66 ± 18.89	28.76 ± 9.79	31.52 ± 11.52
ANSI	Pre	39.95 ± 15.14	46.55 ± 13.63	57.32 ± 10.24	62.10 ± 17.12
	Post	63.76 ± 7.53	71.42 ± 15.20	72.49 ± 6.85	76.79 ± 10.90
SAP	Pre	121 ± 6	120 ± 5	115 ± 10	115 ± 10
	Post	121 ± 9	124 ± 6	115 ± 7	120 ± 10
DAP	Pre	80 ± 5	80 ± 4	80 ± 5	80 ± 2
	Post	85 ± 5	80 ± 1	80 ± 4	80 ± 4

*Note:* The acronym MAD stands for “median absolute deviation”.

**Table 2:** *p*-values of the nonparametric ANOVA-type tests regarding the main effects and interactions of treatments (−55 °C vs. −110 °C), times (T1 vs. T10), and phases (pre vs. post) on the studied variables.

	Main Effects			1st Order Interactions			2nd Order Interaction
Variables	*Treat*	*Time*	*Phase*	*Treat*:*Time*	*Treat*:*Phase*	*Time*:*Phase*	*Treat*:*Time*:*Phase*
RR Mean	0.268	0.433	**<0.001**	0.511	0.728	0.817	0.674
RR TP	0.116	**0.016**	**<0.001**	0.730	0.142	0.385	0.833
RR LFa	0.058	0.140	0.372	0.919	0.579	0.817	0.059
RR HFa	0.077	0.297	**<0.001**	0.246	0.379	0.974	0.769
RR LFnu	0.528	0.595	**0.002**	0.481	0.646	0.749	0.152
RR HFnu	0.767	0.293	**0.014**	0.271	0.828	0.574	0.209
RR LFHF	0.627	0.465	**0.005**	0.465	0.748	0.718	0.127
ΔRRLFnu	0.868	0.578	0.374	0.751	0.337	0.670	0.140
ANSI	0.288	0.121	**<0.001**	0.479	0.988	0.401	0.081
SAP	0.336	0.529	0.371	0.564	0.644	0.709	0.903
DAP	0.450	0.166	0.167	0.683	0.891	0.930	0.815

*Note:* Significant *p*-values are written in bold and coloured in grey.

**Table 3:** Estimated adjusted relative effects and 95% CIs of each time–phase combination computed for the adjusted variables (i.e., the studied variables with values computed as differences with respect to Time = T1 and Phase = Pre).

	Time = T1						Time = T10					
	Phase = Pre			Phase = Post			Phase = Pre			Phase = Post		
Variables	ARE	Lower	Upper	ARE	Lower	Upper	ARE	Lower	Upper	ARE	Lower	Upper
RR Mean	0.272	0.253	0.380	0.728	0.620	0.747	0.417	0.380	0.460	0.582	0.540	0.620
RR TP	0.380	0.310	0.489	0.620	0.511	0.690	0.449	0.395	0.507	0.552	0.493	0.605
RR LFa	0.489	0.392	0.590	0.511	0.411	0.608	0.472	0.412	0.536	0.528	0.464	0.588
RR HFa	0.359	0.296	0.467	0.641	0.533	0.704	0.427	0.373	0.489	0.573	0.511	0.627
RR LFnu	0.576	0.469	0.659	0.424	0.341	0.531	0.571	0.518	0.618	0.429	0.382	0.482
RR HFnu	0.446	0.357	0.551	0.554	0.449	0.643	0.464	0.425	0.505	0.536	0.496	0.575
RR LFHF	0.554	0.449	0.643	0.446	0.357	0.551	0.564	0.495	0.624	0.436	0.376	0.505
ΔRRLFnu	0.424	0.341	0.531	0.576	0.469	0.659	0.490	0.460	0.520	0.510	0.480	0.540
ANSI	0.315	0.271	0.421	0.685	0.579	0.729	0.453	0.425	0.482	0.547	0.518	0.575
SAP	0.489	0.400	0.585	0.511	0.415	0.604	0.462	0.408	0.520	0.538	0.480	0.592
DAP	0.443	0.363	0.538	0.557	0.462	0.637	0.466	0.419	0.515	0.534	0.485	0.581

*Note:* Columns labelled with ARE report the estimated adjusted relative effects ${\hat{p}}_{.ts}$ of the combination (t,s) of the time level t and phase level s, with t=1, 2 and s=1, 2. These effects are marginal because they do not consider the distinction of patients into the two treatment groups. Columns labelled with “lower” and “upper” contain the lower and upper limits, respectively, of the 95% CIs of AREs computed with the procedure in Brunner and Puri [40]. Cells with light grey (Time = T1) or light green (Time = T10) on the background denote nonoverlapping CIs in the pre–post comparison for each variable and time fixed at a specific level. The corresponding AREs then significantly differ at the 0.05 level.

## Data Availability

The dataset used in this study will be uploaded to the Zenodo repository (a link will be provided in case of manuscript acceptance). Requests to access the dataset should be directed to daniela.lucini@unimi.it.

## Supplementary Materials

The following supporting information can be downloaded at: https://www.mdpi.com/article/10.3390/jcm13247728/s1, Supplemental Table S1. Estimated adjusted relative effects (AREs) and 95% confidence intervals of each treatment time–phase combination computed for the adjusted variables (i.e., the studied variables with values computed as differences with respect to Time = T1 and Phase = pre). Supplemental Table S2. Estimated marginal adjusted relative effects (AREs) and 95% confidence intervals of the phase levels computed for the adjusted variables (i.e., the studied variables with values computed as differences with respect to Time = T1 and Phase = pre). Supplemental Table S3. Estimated marginal adjusted relative effects (AREs) and 95% confidence intervals of the time levels computed for the adjusted variables (i.e., the studied variables with values computed as differences with respect to Time = T1 and Phase = pre).

## Abbreviations

a: absolute unit; ANOVA: analysis of variance; ANS: autonomic nervous system; ANSI: Autonomic Nervous System Index; ARE: adjusted relative effect; BMI: body mass index; BAT: brown adipose tissue; CAR: cardiac autonomic regulation; CI: confidence interval; CNS: central nervous system; DAP: diastolic arterial pressure; ECG: electrocardiogram; HF: high-frequency; HR: heart rate; HRV: heart rate variability; IRCCS: Istituto di Ricovero e Cura a Carattere Scientifico; LF: low-frequency; MAD: median absolute deviation; nu: normalized unit; PBC: partial body cryostimulation; PR: percentile rank; RR: RR interval; SAP: systolic arterial pressure; SNS: sympathetic nervous system; TP: total power; WBC: whole-body cold stimulation.
