# Supplementary material for: Whole-Body Cold Stimulation Improves Cardiac Autonomic Control Independently of the Employed Temperature
Source: J Clin Med. 2024 Dec 18;13(24):7728. doi: 10.3390/jcm13247728 (PMC11676992; doi:10.3390/jcm13247728)
Supplement: Supplementary file 1 [file jcm-13-07728-s001.zip › jcm-3332371-supplementary.pdf]

Whole-Body Cold Stimulation Improves Cardiac Autonomic Control Independently of the Employed Temperature
Supplementary Information

Supplemental Table S1. Estimated adjusted relative effects (AREs) and 95% confidence intervals of each treatment time–phase combination computed for the adjusted variables (i.e., the studied variables with values computed as differences with respect to Time=T1 and Phase=pre)

RR Mean (profile plot in Fig. 2)

Treatment	Time–Phase	ARE	Lower	Upper
−55°C	T1–pre	0.2663	0.2020	0.3520
−55°C	T10–pre	0.4786	0.3524	0.6089
−55°C	T1–post	0.6047	0.5085	0.6904
−55°C	T10–post	0.6605	0.5602	0.7430
−110°C	T1–pre	0.2663	0.1999	0.3495
−110°C	T10–pre	0.4212	0.2054	0.6806
−110°C	T1–post	0.6562	0.5173	0.7687
−110°C	T10–post	0.6372	0.3917	0.8184

RR TP (profile plot in Fig. 2)

Treatment	Time–Phase	ARE	Lower	Upper
−55°C	T1–pre	0.3315	0.2363	0.4534
−55°C	T10–pre	0.4518	0.3339	0.5781
−55°C	T1–post	0.4888	0.3807	0.5984
−55°C	T10–post	0.5366	0.4085	0.6582
−110°C	T1–pre	0.3315	0.2338	0.4512
−110°C	T10–pre	0.6277	0.4671	0.7600
−110°C	T1–post	0.6658	0.4838	0.8022
−110°C	T10–post	0.7337	0.4859	0.8751

RR LFa

Treatment	Time–Phase	ARE	Lower	Upper
−55°C	T1–pre	0.4511	0.3388	0.5710
−55°C	T10–pre	0.4207	0.2985	0.5587
−55°C	T1–post	0.3895	0.2748	0.5254
−55°C	T10–post	0.5692	0.4503	0.6776
−110°C	T1–pre	0.4511	0.3378	0.5709
−110°C	T10–pre	0.6345	0.3907	0.8158
−110°C	T1–post	0.6658	0.4257	0.8325
−110°C	T10–post	0.5666	0.3237	0.7762

RR HFa

Treatment	Time–Phase	ARE	Lower	Upper
−55°C	T1–pre	0.3315	0.2501	0.4316
−55°C	T10–pre	0.4830	0.3544	0.6148
−55°C	T1–post	0.5083	0.3901	0.6252
−55°C	T10–post	0.6641	0.5698	0.7421
−110°C	T1–pre	0.3315	0.2484	0.4300
−110°C	T10–pre	0.4185	0.2059	0.6752
−110°C	T1–post	0.6943	0.5019	0.8281
−110°C	T10–post	0.5802	0.3335	0.7865

RR LFnu (profile plot in Fig. 2)

Treatment	Time–Phase	ARE	Lower	Upper
−55°C	T1–pre	0.6576	0.5498	0.7455
−55°C	T10–pre	0.4670	0.3407	0.5997
−55°C	T1–post	0.4344	0.3091	0.5731
−55°C	T10–post	0.3417	0.2540	0.4492
−110°C	T1–pre	0.6576	0.5514	0.7474
−110°C	T10–pre	0.5747	0.3583	0.7614
−110°C	T1–post	0.5666	0.3417	0.7627
−110°C	T10–post	0.3872	0.2160	0.6006

RR HFnu

Treatment	Time–Phase	ARE	Lower	Upper
−55°C	T1–pre	0.3533	0.2616	0.4644
−55°C	T10–pre	0.5786	0.4572	0.6877
−55°C	T1–post	0.5178	0.3732	0.6582
−55°C	T10–post	0.6351	0.5299	0.7242
−110°C	T1–pre	0.3533	0.2597	0.4629
−110°C	T10–pre	0.4090	0.2155	0.6437
−110°C	T1–post	0.4823	0.2787	0.6933
−110°C	T10–post	0.5965	0.3982	0.7627

RR LFHF

Treatment	Time–Phase	ARE	Lower	Upper
−55°C	T1–pre	0.6359	0.5296	0.7256
−55°C	T10–pre	0.4562	0.3340	0.5864
−55°C	T1–post	0.4428	0.3080	0.5908
−55°C	T10–post	0.3793	0.2659	0.5161
−110°C	T1–pre	0.6359	0.5308	0.7272
−110°C	T10–pre	0.5842	0.3537	0.7775
−110°C	T1–post	0.5645	0.3430	0.7589
−110°C	T10–post	0.3764	0.2143	0.5809

ΔRRLFnu

Treatment	Time–Phase	ARE	Lower	Upper
−55°C	T1–pre	0.4076	0.2948	0.5366
−55°C	T10–pre	0.5598	0.4410	0.6695
−55°C	T1–post	0.5982	0.4785	0.7030
−55°C	T10–post	0.5736	0.4404	0.6929
−110°C	T1–pre	0.4076	0.2930	0.5358
−110°C	T10–pre	0.4497	0.2476	0.6740
−110°C	T1–post	0.4117	0.2449	0.6072
−110°C	T10–post	0.4701	0.2683	0.6843

ANSI (profile plot in Fig. 2)

Treatment	Time–Phase	ARE	Lower	Upper
−55°C	T1–pre	0.2989	0.2066	0.4263
−55°C	T10–pre	0.5641	0.4370	0.6802
−55°C	T1–post	0.6178	0.4994	0.7186
−55°C	T10–post	0.6264	0.4728	0.7499
−110°C	T1–pre	0.2989	0.2033	0.4230
−110°C	T10–pre	0.4171	0.2478	0.6138
−110°C	T1–post	0.5448	0.4231	0.6605
−110°C	T10–post	0.5380	0.3570	0.7078

SAP

Treatment	Time–Phase	ARE	Lower	Upper
−55°C	T1–pre	0.4830	0.3639	0.6049
−55°C	T10–pre	0.5015	0.3798	0.6230
−55°C	T1–post	0.5205	0.4051	0.6327
−55°C	T10–post	0.6242	0.4792	0.7421
−110°C	T1–pre	0.4830	0.3630	0.6053
−110°C	T10–pre	0.3336	0.1624	0.5815
−110°C	T1–post	0.4911	0.3282	0.6563
−110°C	T10–post	0.4156	0.2025	0.6740

DAP

Treatment	Time–Phase	ARE	Lower	Upper
−55°C	T1–pre	0.5057	0.3934	0.6171
−55°C	T10–pre	0.4303	0.3003	0.5762
−55°C	T1–post	0.6080	0.4715	0.7232
−55°C	T10–post	0.5443	0.4010	0.6776
−110°C	T1–pre	0.5057	0.3930	0.6177
−110°C	T10–pre	0.3555	0.1947	0.5681
−110°C	T1–post	0.5487	0.3426	0.7368
−110°C	T10–post	0.4010	0.1842	0.6763

Supplemental Table S2. Estimated marginal adjusted relative effects (AREs) and 95% confidence intervals of the phase levels computed for the adjusted variables (i.e., the studied variables with values computed as differences with respect to Time=T1 and Phase=pre)

	Phase: Pre			Phase: Post		
**Variables**	**ARE**	**Lower**	**Upper**	**ARE**	**Lower**	**Upper**
RR Mean[a]	0.3568	0.3259	0.3959	0.6432	0.6041	0.6741
RR TP[a]	0.4371	0.3786	0.5042	0.5629	0.4958	0.6214
RR LFa	0.4967	0.4318	0.5620	0.5033	0.4380	0.5682
RR HFa	0.4116	0.3592	0.4747	0.5884	0.5253	0.6408
RR LFnu[a]	0.5789	0.5175	0.6313	0.4211	0.3687	0.4825
RR HFnu	0.4447	0.3933	0.5015	0.5553	0.4985	0.6067
RR LFHF	0.5534	0.4909	0.6096	0.4466	0.3904	0.5091
ΔRRLFnu	0.4825	0.4392	0.5269	0.5175	0.4731	0.5608
ANSI[a]	0.4116	0.3749	0.4533	0.5884	0.5467	0.6251
SAP	0.4473	0.3895	0.5117	0.5527	0.4883	0.6105
DAP	0.4489	0.4024	0.4993	0.5511	0.5007	0.5976

Note: Columns labelled with ARE report the estimated marginal adjusted relative effects $\hat{p}_{..s}$ of the phase level s, with $s = 1, 2$. Columns labelled with "Lower" and "Upper" contain the lower and upper limits, respectively, of the 95% confidence intervals (CIs) of AREs. Cells with light grey on the background denote nonoverlapping CIs in the pre–post comparison for each variable. The corresponding AREs then significantly differ at the 0.05 level.

[a]Data reported in the panels of Fig. 2.

Supplemental Table S3. Estimated marginal adjusted relative effects (AREs) and 95% confidence intervals of the time levels computed for the adjusted variables (i.e., the studied variables with values computed as differences with respect to Time=T1 and Phase=pre)

	Time: T1			Time: T10		
**Variables**	**ARE**	**Lower**	**Upper**	**ARE**	**Lower**	**Upper**
RR Mean[a]	0.4674	0.3874	0.5549	0.5326	0.4451	0.6126
RR TP[a]	0.4447	0.4026	0.4904	0.5553	0.5096	0.5974
RR LFa	0.4825	0.4161	0.5515	0.5175	0.4485	0.5839
RR HFa	0.4457	0.3845	0.5145	0.5543	0.4855	0.6155
RR LFnu[a]	0.5458	0.4685	0.6151	0.4542	0.3849	0.5315
RR HFnu	0.4494	0.3825	0.5249	0.5506	0.4751	0.6175
RR LFHF	0.5080	0.4298	0.5847	0.4920	0.4153	0.5702
ΔRRLFnu	0.4636	0.3993	0.5333	0.5364	0.4667	0.6007
ANSI[a]	0.4438	0.3691	0.5308	0.5562	0.4692	0.6309
SAP	0.4664	0.3942	0.5448	0.5336	0.4552	0.6058
DAP	0.5615	0.4776	0.6328	0.4385	0.3672	0.5224

Note: Columns labelled with ARE report the estimated marginal adjusted relative effects $\hat{p}_{.t.}$ of the time level t, with $t = 1, 2$. Columns labelled with "Lower" and "Upper" contain the lower and upper limits, respectively, of the 95% confidence intervals (CIs) of AREs. Cells with light green on the background denote nonoverlapping CIs in the T1–T10 comparison for each variable. The corresponding AREs then significantly differ at the 0.05 level.

[a]Data reported in the panels of Fig. 2.
